# Social Inequalities in Prenatal Folic Acid Supplementation: Results from the ELFE Cohort

**DOI:** 10.3390/nu11051108

**Published:** 2019-05-18

**Authors:** Aurore Camier, Manik Kadawathagedara, Sandrine Lioret, Corinne Bois, Marie Cheminat, Marie-Noëlle Dufourg, Marie Aline Charles, Blandine de Lauzon-Guillain

**Affiliations:** 1 INSERM, UMR1153 Center for Research in Epidemiology and StatisticS (CRESS), Research Team on Early Life Origins of Health (EAROH), 75004 Paris, France; aurore.camier@inserm.fr (A.C.); manik.kadawathagedara@inserm.fr (M.K.); sandrine.lioret@inserm.fr (S.L.); marie-aline.charles@inserm.fr (M.A.C.); 2 Université de Paris, UMR1153 Center for Research in Epidemiology and StatisticS (CRESS), Research Team on Early Life Origins of Health (EAROH), 75004 Paris, France; 3 Unité Mixte Inserm-Ined-EFS Elfe, Ined, 75020 Paris, France; corinne.bois@ined.fr (C.B.); marie.cheminat@ined.fr (M.C.); marie-noelle.dufourg@ined.fr (M.-N.D.); 4 INRA, U1125 Center for Research in Epidemiology and StatisticS (CRESS), Research Team on Early Life Origins of Health (EAROH), 75004 Paris, France

**Keywords:** folic acid supplementation, pregnancy, epidemiology, social inequalities

## Abstract

Most professional and international organizations recommend folic acid supplementation for women planning pregnancy. Various studies have shown high levels of non-compliance with this recommendation. This study aimed to identify sociodemographic characteristics related to this compliance. The analyses were based on 16,809 women from the French nationwide ELFE cohort (Etude Longitudinale Française depuis l’Enfance). Folic acid supplementation was assessed at delivery, and sociodemographic characteristics were collected at two months postpartum. The association between sociodemographic characteristics and compliance with recommendations on folic acid supplementation (no supplementation, periconceptional supplementation, and supplementation only after the periconceptional period) was examined using multivariate multinomial logistic regression. Only 26% of French women received folic acid supplementation during the periconceptional period, 10% of women received supplementation after the periconceptional period, and 64% received no supplementation. Young maternal age, low education level, low family income, multiparity, single parenthood, maternal unemployment, maternal overweight, and smoking during pregnancy were related to lower likelihood of folic acid supplementation during the periconceptional period compared to no supplementation. These associations were not explained by unplanned pregnancy. Immigrant and underweight women were more likely to receive folic acid supplementation after the periconceptional period. Our study confirms great social disparities in France regarding the compliance with the recommendations on folic acid supplementation.

## 1. Introduction

Neural tube defects are one of the most common congenital diseases in Europe [1] and, in France, represent 1.3 cases (live births, fetal deaths, termination of pregnancy for fetal anomaly) per 1000 births [2]. Randomized trials have proved the efficacy of folic acid supplementation during the periconceptional period in neural tube defects prevention [3,4,5].

Most professional and international organizations, including the World Health Organization, recommend folic acid supplementation for women planning pregnancy [6,7]. However, the specifically targeted population groups (e.g., women of childbearing age, women planning pregnancy, women of childbearing age without safe contraception) and the timing or duration of the supplementation recommendations vary across countries. In North America, maternal folic acid intake is addressed by the folic acid fortification of food products (flours, cereals) [8], whereas, in Europe, the prevention policy is based on supplementation exclusively. In France, the latest guidelines from the Agency for Food, Environmental, and Occupational Health & Safety reinforced the need for folic acid supplementation for women within the periconceptional period (eight weeks before and eight weeks after conception) to achieve a daily intake of 400 μg dietary folate equivalents [9].

The neural tube defects prevention policy based on folic acid supplementation during the periconceptional period assumes that pregnancies are planned and that future mothers visit health professionals before pregnancy. However, in Europe, 45% of pregnancies were unintended in 2012 [10], and women from disadvantaged households tend to have lower access to health services, particularly for preventive care [11,12,13].

Within this context, the objective of the present study was to identify sociodemographic characteristics associated with compliance with recommendations on folic acid supplementation to prevent neural tube defects in France, with a special focus on the timing of this supplementation, on the basis of a national survey performed in 2011 on women giving birth.

## 2. Materials and Methods

### 2.1. Study Population

The present study was based on the ELFE study (Etude Longitudinale Française depuis l’Enfance), a multidisciplinary study comprising a nationally representative birth cohort, which included 18,258 children born in 349 randomly selected maternity units in France in 2011 [14]. Inclusion took place during 25 selected recruitment days over 4 waves encompassing 4 to 8 days each and all 4 seasons. Inclusion criteria were as follows: children born after 33 weeks of gestation, mothers aged 18 years or older and who were not planning to move outside of metropolitan France during the following three years. Foreign families could also participate in the study if mothers were able to read French, Arabic, Turkish, or English. Participating mothers signed informed consent for themselves and their child. A total of 51% of contacted parents agreed to participate. Data were collected through standardized interviews conducted by trained midwives and through self-completed questionnaires. The follow-up of this birth cohort is ongoing.

The ELFE study was given ethical approval by the Advisory Committee for the Treatment of Information on Health Research (Comité Consultatif sur le Traitement des Informations pour la Recherche en Santé), the National Agency Regulating Data Protection (Commission National Informatique et Libertés), and the National Statistics Council.

### 2.2. Maternal Characteristics

Mothers were first interviewed in the maternity ward after delivery, to collect information about their pregnancy, their newborn, and their general characteristics (employment status, education level, age). Two months post-partum, telephone interviews with mothers and fathers took place, which included additional questions on demographic and socioeconomic characteristics such as country of birth, educational level, employment, monthly income, and number of family members.

As family data were more comprehensively collected during the two-month-post-partum interview than during the maternity interview and because family sociodemographic characteristics only marginally evolved within two months, we prioritized data collected at two months in our analyses. The sociodemographic characteristics collected during the maternity stay were used only in the case of missing values in the two-months-post-partuminterview. Maternal characteristics included in the analyses were: migration status (native French, immigrant, descendant of immigrants), age at first delivery (18–25 years, 25–29 years, 30–34 years, ≥35 years), family type (traditional, stepfamily, one-parent family), educational level measured on the basis of the highest academic degree attained (<secondary school, secondary school, high school, two-year university degree, ≥three-year university degree), employment status (employed, housewife/parental leave, retired/disability/unemployed, student, other), monthly family income (≤€1500, €1501–2300, €2301–3000, €3001–4000, €4001–5000, >€5000), smoking status (never smoked, smoked only before pregnancy, smoked until early pregnancy, smoked during the whole pregnancy), planned pregnancy, and fertility treatment.

### 2.3. Folic Acid Supplementation

Information on maternal folic acid supplementation was collected retrospectively at the maternity unit during face-to-face interviews utilizing five questions: ‘Have you taken folic acid (also called vitamin B9) before and/or during pregnancy (to prevent nervous system abnormalities)?’ ‘If yes, indicate the periods of time you took it: one to three months before pregnancy (yes/no), in the first two months of pregnancy (yes/no), between the second and sixth month of pregnancy (yes/no), beyond six months of pregnancy (yes/no)’. Women were divided into three groups according to their folic acid supplementation: supplementation during the periconceptional period (before pregnancy and/or first two months of pregnancy), late supplementation (only after the second month of pregnancy), and no supplementation. No information about family history of neural tube defects or folic acid supplements dosages was collected.

### 2.4. Sample Selection

Women who withdrew consent within the first year (*n* = 128) or for whom it was not possible to verify the eligibility criteria due to missing data (*n* = 350) were excluded from the study, resulting in 17,574 eligible mothers. We also excluded women with missing data (*n* = 3418), leaving a total of 14,156 women in the main analyses.

### 2.5. Statistical Analyses

Comparisons between excluded and included subjects were conducted with chi-square tests.

In order to provide representative descriptive statistics of births in 2011 in France, the descriptive data (rates) were weighted to consider the sampling design and biases related to non-consent. Weighting also included calibration on margins from the state register of statistical data and the 2010 French National Perinatal study [15] regarding the following variables: age, region, marital status, migration status, level of education, and primiparity.

Associations between sociodemographic variables and folic acid supplementation were tested by multivariable multinomial logistic regression, including maternal characteristics (maternal age at first delivery, parity, family composition, migration status, education level, employment status, family income, pre pregnancy body mass index (BMI), smoking status), and additionally adjusted for mothers’ region of residence, size of maternity unit, and wave of recruitment.

Several sensitivity analyses were performed. First, analyses were performed only for women with planned pregnancy and without fertility treatment, in order to check that the associations between familial characteristics and compliance with folic acid supplementation guidelines were not driven by the specific cases of unplanned pregnancy or fertility treatment. Then, we calculated weighted multivariate models. Afterward, using multiple imputations to deal with missing data on sociodemographic variables, using the SAS software. We assumed that data were missing at random and generated five independent datasets using the fully conditional specification method (MI procedure, FCS statement, NIMPUTE option) and then calculated pooled effect estimates (MIANALYSE procedure). Imputation model variables included both the potentially predicting non-response and the outcomes. Categorical variables were imputed using a multinomial model, ordinal or binary variables using logistic regressions, and continuous variables using linear regressions. Further details are available in Supplementary Table S1.

## 3. Results

Women excluded from our analysis were younger, more likely to be single, born in a country other than France, had a lower education level, were less likely to be employed during pregnancy, and had quit smoking before pregnancy compared to women included in our analysis (Table 1).

The weighted rate of folic acid supplementation was 26.0% during the periconceptional period and 9.9% for supplementation started only after the first two months of pregnancy. A total of 64.1% of women did not receive folic acid supplementation before or during pregnancy.

Bivariable associations between maternal characteristics and folic acid supplementation are shown in Table 2.

Women were less likely to take folic acid supplementation during the periconceptional period, compared to no pre-conceptional supplementation, when they were younger, multiparous, single, with low education level, low family income, unemployed during pregnancy, and smoked during pregnancy (Table 3). Compared to mothers who had never smoked, women who had quit smoking before pregnancy were more likely to take folic acid supplementation during the periconceptional period, whereas the relationship was inverse for those women who smoked during the whole pregnancy.

Women were more likely to start folic acid supplementation after the periconceptional period when they were older, underweight before pregnancy, or immigrant.

The results were very similar when the specific weighting to deal with non-inclusion bias was used in sensitivity analyses (Supplementary Table S2). Moreover, the results were not modified by excluding women with an unplanned pregnancy and those with fertility treatment. In the analysis by multiple imputation with five independent datasets, the findings were very consistent with those described in our main analysis.

## 4. Discussion

The results from our study revealed that only 26% of French pregnant women received folic acid supplementation during the periconceptional period. Moreover, we found that all dimensions related to the socioeconomic level (young maternal age, low education level, employment status, income, and single parenthood) were independently related to a lower odds ratio of pre-conceptional folic acid supplementation. These associations remained after excluding women with an unplanned pregnancy or those who had received fertility treatment.

In most countries in Europe, the rates of periconceptional supplementation are still low, even though guidelines on this public health issue were introduced more than 15 years ago. Most countries report folic acid supplementation rates of 25–35% during the periconceptional period [16]. In the Dutch Generation R cohort, 37% of women received folic acid supplementation within the appropriate period [17]. The results were lower in Norway and Denmark, with 10% and 14% of women receiving folic acid supplementation during the periconceptional period, respectively [18,19]. In France, the results from the 2010 National Perinatal Survey indicated that 24% of women received folic acid supplementation in the periconceptional period [20], which is consistent with the rate highlighted in the present study. This low compliance with the folic acid supplementation guidelines could impair the effectiveness of the current neural tube defects prevention policy, and some studies have indicated that the implementation of the recommendations on folic acid supplementation is not clearly related to a decrease in the incidence of neural tube defects [21,22,23].

Folic acid supplementation rates vary according to country, but sociodemographic variables such as younger age of women and lower family income have consistently been associated with lower rates of folic acid supplementation [17,18,24,25,26]. European supplementation policies similar to those of France, without fortification policies, might, therefore, play a role in maintaining social inequalities [27,28]. In our study, social inequalities in periconceptional folic acid supplementation remained even when pregnancy planning was considered. This finding accentuates the poor preventive care during early pregnancy of the most socially disadvantaged women. The main reasons may be a decreased awareness in disadvantaged populations regarding this recommendation as well as their lower use of health care facilities [29,30]. To address this issue in the Netherlands, a mass media campaign was implemented in 1995 to increase the rate of folic acid supplementation [30].

Interestingly, in our study, women who stopped smoking before pregnancy were more likely to have folic acid supplementation during the periconceptional period, suggesting that they were probably more inclined to follow guidelines related to pregnancy and be involved in preventive care. This situation may give some leads for further folic acid supplementation strategies, such as considering the preventive prenatal care as a specific moment to address women’s general health (nutrition, lifestyle, physical activity, smoking cessation).

Preventing neural tube defects by folic acid supplementation is a difficult goal to achieve, as changes in behavior are difficult to generate, and the human reproduction period can be long [31]. Indeed, the time between planning a pregnancy and becoming pregnant may take several months or even years, and women have first to achieve and maintain the objective of acid folic intake. To address this issue, more than 40 countries have decided to fortify foods with folic acid, and this strategy appears to be the most effective today [8,23,29,32]. This strategy could be particularly relevant for young and low-income women, because these groups of women are more likely to have unplanned pregnancies and less likely to receive or respond to health-promotion messages [16]. Studies in Canada suggest an improved efficacy of food fortification policies: while no effect on the incidence of neural tube defects was observed after recommendations of folic acid periconceptional supplementation between 1993 and 1997, a marked decrease in the incidence of neural tube defects was observed after food fortification implementation in 1997 [8,23]. The target group of this fortification policy are women of childbearing age, even though the general population has increased dietary folate intake [29]. This fortification strategy aims to supplement women continuously during their reproductive period, reaching women with unplanned pregnancies, and to limit social inequalities in health [8,33]. In 2017, a recommendation by the US Preventive Services Task Force (USPSTF) reinforced the importance of folic acid supplementation for all women of childbearing age (even in the absence of planned pregnancies) in addition to food fortification [34]. One of the disadvantages of this strategy concerns elderly people. Indeed, high folate intake may mask anemia resulting from vitamin B12 deficiency, which may cause neurological deterioration [35]. Moreover, there is a supposed relation between high intake of folic acid and cancer risk (especially colorectal cancer), even though human epidemiological data are inconclusive [36]. Even if voluntary fortification is very common in Europe (e.g., breakfast cereals, dairy products, fruit juices), mandatory food fortification is not practiced in any European countries mainly because of this cancer risk [36]. In France, systematic flour fortification with folic acid was considered. A pilot study to assess risks and benefits was proposed in early 2000s but ultimately was not launched. Our findings could contribute to this debate [37].

Some maternal characteristics, such as immigrant status and pre-pregnancy underweight status, were specifically related to folic acid supplementation only after the periconceptional period. This could be due to the fact that this late supplementation is not taken for the prevention of neural tube defects but is started following treatment for other pregnancy issues. The WHO recommends daily oral iron and folic acid supplementation among pregnant women to prevent maternal anaemia, puerperal sepsis, low birth weight, and preterm birth [38]. In the ELFE study, women who had started folic acid supplementation only after the periconceptional period were more likely to have haemoglobin levels below 11 g/100 mL during pregnancy (15% vs 20%, *p* < 0.0001), compared to women who received folic acid supplementation during the periconceptional period. Immigrant and underweight women are probably more at risk of maternal anaemia [39] and low birth weight.

The ELFE cohort is a representative study of births from the year 2011 in metropolitan France (excluding very premature babies), and descriptive statistics used weighted data to provide accurate prevalence of folic acid supplementation. In our multivariate analysis of the association between familial characteristics and folic acid supplementation, we excluded part of the sample because of missing data. The comparison of the characteristics of the women included versus those of the women excluded from the analysis showed that women with a higher educational level were overrepresented in the study, which may have implications for generalizing the findings. However, in sensitivity analyses based on multiple imputations of missing data, the results remained similar. Because women were recruited at delivery, it is necessary to acknowledge that miscarriages or medical abortions due to congenital malformations related to folic acid condition were not included in the present study. The main strengths of the ELFE study include the large sample size and the wide range of sociodemographic variables, which allowed us to demonstrate that various components of the social background were related to compliance with folic acid supplementation guidelines. Unfortunately, we were not able to distinguish between the timing patterns of folic acid supplementation related to neural tube defects prevention from supplementation for other purposes. Moreover, as we did not record the dose of folic acid consumed by women, we were not able to identify women with folic acid intake higher than the tolerable upper limit of 1000 μg/day [40]. Finally, the study design did not allow to assess the effect of such supplementation on birth outcomes as stillbirth, very premature birth, and termination of pregnancy.

## 5. Conclusions

Our study confirms low rates of folic acid supplementation during the periconceptional period as well as great social disparities concerning the use of maternal periconceptional folic acid supplementation. Therefore, it is important to find alternative methods, especially for vulnerable populations, to increase the effectiveness of prevention policies by considering media campaign.

## Figures and Tables

**Table 1:** Study sample characteristics.

	Excluded Women	Included Women	*p*-Value
N	3418	14,156	
Age at delivery (years)	29.4 (5.7)	30.4 (4.9)	<0.0001
Birth order			<0.0001
First child	43.7% (1491)	45.0% (6365)	
Second child	33.5% (1142)	36.0% (5101)	
Third child	14.3% (488)	13.8% (1949)	
Fourth child or more	8.5% (291)	5.2% (741)	
Single parenthood			<0.0001
Yes	12.4% (414)	3.9% (548)	
No	87.6% (2928)	96.1% (13,549)	
Country of birth			<0.0001
France	78.5% (2599)	88.9% (12,516)	
Another country	21.5% (713)	11.1% (1568)	
Education level			<0.0001
Primary school	11.2% (376)	3.5% (491)	
Secondary school	20.0% (673)	12.6% (1783)	
General high school	11.2% (378)	7.2% (1023)	
Technical/professional high school	15.7% (530)	12.3% (1741)	
University	41.9% (1410)	64.4% (9116)	
Employment status			<0.0001
Employed	59.3% (1968)	72.7% (10,298)	
Housewife/parental leave	2.5% (83)	3.4% (485)	
Retired/disability/unemployed	11.9% (395)	12.0% (1702)	
Student	21.7% (721)	10.1% (1424)	
Other	4.6% (154)	1.7% (247)	
Pre-pregnancy body mass index (kg/m^2^)	23.7 (5.1)	23.4 (4.7)	0.0138
Smoking status			<0.0001
Never smoked	59.0% (1902)	56.7% (8020)	
Smoked only before pregnancy	18.1% (585)	23.7% (3356)	
Smoked only during early pregnancy	4.0% (130)	3.9% (559)	
Smoker during the whole pregnancy	18.9% (608)	15.7% (2221)	

Values are % (*n*).

**Table 2:** Bivariate analyses between familial characteristics and folic acid supplementation.

	*N*	No Supplementation (*N* = 8328)	Periconceptional Supplementation (*N* = 4520)	Late Supplementation Only (*N* = 1308)	*p*-Value
Age at first delivery					<0.0001
<25 years	3611	74.9%	15.1%	9.6%	
25–29 years	6134	59.9%	31.3%	8.7%	
30–34 years	3412	50.7%	38.7%	9.8%	
≥35 years	999	49.9%	39.3%	9.6%	
Parity					<0.0001
First child	6365	55.2%	35.4%	9.0%	
Second child	5101	63.4%	26.9%	9.3%	
Third child	1949	67.9%	21.8%	9.7%	
Fourth child or more	741	78.4%	10.9%	10.1%	
Family composition					<0.0001
Traditional	12,499	59.7%	30.6%	9.2%	
Single parenthood	532	80.7%	11.1%	8.8%	
Stepfamily	1125	68.8%	20.9%	10.3%	
Migration					<0.0001
Native French	10,229	60.9%	30.1%	8.7%	
First-generation immigrant	1582	64.0%	23.5%	11.7%	
Second-generation immigrant	2345	61.7%	27.9%	9.7%	
Educational level					<0.0001
<Secondary school	999	76.9%	11.8%	10.1%	
Secondary school	2010	75.3%	16.3%	8.9%	
High school	2620	66.4%	23.9%	8.9%	
Two-year university degree	3199	58.6%	31.8%	9.5%	
Three-year university degree	2495	52.3%	37.8%	9.4%	
≥Five-year university degree	2833	46.5%	43.4%	9.0%	
Employment status					<0.0001
Employed	10,298	57.0%	33.7%	9.0%	
Student	485	61.8%	30.4%	8.7%	
Unemployed	1702	68.5%	20.6%	11.2%	
Housewife/parental leave	1424	74.6%	13.9%	9.6%	
Other	247	71.8%	21.9%	6.9%	
Family income					<0.0001
<€1500/month	1417	78.4%	12.3%	9.2%	
€1501–2300/month	2177	69.3%	19.5%	10.5%	
€2301–3000/month	3974	61.4%	29.3%	9.1%	
€3001–4000/month	3745	56.6%	33.8%	9.2%	
€4001–5000/month	1551	50.0%	40.1%	9.1%	
>€5000/month	1292	45.5%	45.9%	8.0%	
Smoking status					<0.0001
Never smoked	8020	59.7%	30.5%	9.3%	
Smoked only before pregnancy	3356	56.7%	33.7%	9.0%	
Smoked only during early pregnancy	559	68.8%	21.6%	10.7%	
Smoked during the whole pregnancy	2221	72.2%	18.3%	9.1%	
Planned pregnancy					<0.0001
No	1203	74.6%	15.3%	10.1%	
Yes	12,079	58.8%	31.6%	9.0%	
Previous treatment for infertility					<0.0001
No	12,915	63.0%	27.2%	9.4%	
Yes	1105	42.4%	49.6%	7.4%	

Values are *n* and weighted %. Chi-square tests on weighted data.

**Table 3:** Multivariate associations between sociodemographic characteristics and timing of folic acid supplementation (*n* = 14,156).

	Folic Acid Supplementation (Reference = No Supplementation)		
	Periconceptional Supplementation	Late Supplementation Only	*p*-Value
Age at first delivery			<0.0001
<25 years	0.72 [0.64–0.81]	1.04 [0.88–1.23]	
25–29 years	1 [Ref]	1 [Ref]	
30–34 years	1.23 [1.12–1.36]	1.32 [1.13–1.54]	
≥35 years	1.30 [1.12–1.51]	1.33 [1.04–1.71]	
Birth order			<0.0001
First child	1 [Ref]	1 [Ref]	
Second child	0.62 [0.57–0.68]	0.92 [0.80–1.05]	
Third child	0.58 [0.51–0.66]	0.96 [0.80–1.17]	
Fourth child or more	0.40 [0.32–0.51]	0.87 [0.66–1.16]	
Family composition			<0.0001
Traditional	1 [Ref]	1 [Ref]	
Single-parenthood	0.73 [0.56–0.96]	0.80 [0.57–1.12]	
Stepfamily	1.22 [1.04–1.43]	1.21 [0.97–1.51]	
Migration			<0.0001
Native French	1 [Ref]	1 [Ref]	
Immigrant	0.90 [0.79–1.04]	1.31 [1.08–1.59]	
Descendant of immigrant	0.92 [0.83–1.03]	1.07 [0.90–1.26]	
Education level			<0.0001
<Secondary school	0.47 [0.38–0.59]	0.73 [0.54–0.98]	
Secondary school	0.51 [0.43–0.61]	0.67 [0.52–0.87]	
High school	0.68 [0.60–0.79]	0.74 [0.59–0.93]	
Two-year university degree	0.76 [0.68–0.86]	0.89 [0.73–1.09]	
Three-year university degree	0.89 [0.79–1.00]	0.90 [0.74–1.11]	
≥Five-year university degree	1 [Ref]	1 [Ref]	
Employment status			<0.0001
Employed	1 [Ref]	1 [Ref]	
Retired/disability/unemployed	0.86 [0.75–0.98]	1.20 [1.00–1.45]	
Housewife/parental leave	0.82 [0.69–0.97]	0.93 [0.74–1.16]	
Other	0.84 [0.62–1.14]	0.64 [0.38–1.07]	
Student	1.11 [0.90–1.37]	1.00 [0.71–1.41]	
Monthly family income			<0.0001
<€1500	0.62 [0.51–0.75]	0.89 [0.69–1.14]	
€1501–2300	0.77 [0.68–0.89]	1.06 [0.87–1.28]	
€2301–3000	1 [Ref]	1 [Ref]	
€3001–4000	1.05 [0.94–1.16]	1.03 [0.87–1.22]	
€4001–5000	1.10 [0.95–1.26]	0.98 [0.77–1.23]	
>€5000	1.32 [1.13–1.54]	0.91 [0.69–1.19]	
Pre-pregnancy body mass index			<0.0001
<18.5 kg/m^2^	1.06 [0.91–1.23]	1.31 [1.05–1.63]	
18.5–24.9 kg/m^2^	1 [Ref]	1 [Ref]	
25.0–29.9 kg/m^2^	0.79 [0.71–0.88]	0.93 [0.79–1.10]	
30 kg/m^2^ or more	0.72 [0.62–0.84]	0.96 [0.78–1.18]	
Smoking status			<0.0001
Never smoked	1 [Ref]	1 [Ref]	
Only before pregnancy	1.10 [1.01–1.21]	1.04 [0.89–1.21]	
Only during early pregnancy	0.72 [0.58–0.88]	1.09 [0.82–1.47]	
During the whole pregnancy	0.68 [0.60–0.77]	0.89 [0.74–1.07]	

Values are adjusted OR [95% CI]. Multivariate multinomial logistic regression, also adjusted for maternal region of residence, size of maternity unit, and wave of recruitment.

## Supplementary Materials

The following are available online at https://www.mdpi.com/2072-6643/11/5/1108/s1, Table S1: Type of variable, model used to predict missing data, and percentage of values missing for each variable included in the imputation model, Table S2: Sensitivity analyses on multivariate associations between familial characteristics and timing of folic acid supplementation in comparison to no supplementation
