# Supplementary material for: Social Inequalities in Prenatal Folic Acid Supplementation: Results from the ELFE Cohort
Source: Nutrients. 2019 May 18;11(5):1108. doi: 10.3390/nu11051108 (PMC6566921; doi:10.3390/nu11051108)
Supplement: Supplementary file 1 [file nutrients-11-01108-s001.pdf]

SOCIAL INEQUALITIES IN PERINATAL FOLIC ACID SUPPLEMENTATION: RESULTS FROM THE ELFE COHORT
# – ONLINE SUPPLEMENTAL MATERIAL -

Supplementary Table 1. Type of variable, model used to predict missing data, and percentage of values missing for each variable included in the imputation model (n=16,802).

Variable	Type of variable	Model used to predict missing data	Percentage of missing values
Timing of folic acid supplementation	Categorical (3 categories)	No missing data	0.0%
Maternal age at delivery	Continuous	No missing data	0.0%
Maternity size	Categorical (5 categories)	No missing data	0.0%
Maternity unit's category	Categorical (3 categories)	No missing data	0.0%
Random maternity number	Continuous	No missing data	0.0%
Primiparous women	Binary	No missing data	0.0%
Recruitment wave	Categorical (4 categories)	No missing data	0.0%
Region	Categorical (9 categories)	No missing data	0.0%
Educational level (collected at delivery)	Ordinal (5 categories)	Logistic regression	0.0%
Parity	Continuous	Linear regression	0.0%
Single parenthood	Binary	Logistic regression	0.5%
Employment status	Categorical (5 categories)	Multinomial regression	0.5%
Country of birth	Binary	Logistic regression	0.7%
Smoking status	Categorical (4 categories)	Multinomial regression	0.8%
Treatment for infertility	Binary	Logistic regression	1.1%
Pre-pregnancy BMI	Continuous	Linear regression	1.2%
Age at first delivery	Continuous	Linear regression	6.0%
Family composition	Categorical (3 categories)	Multinomial regression	10.0%
Educational level (collected at 2-months post-partum)	Ordinal (6 categories)	Logistic regression	10.7%
Migration	Categorical (3 categories)	Multinomial regression	12.9%
Family income	Ordinal (6 categories)	Logistic regression	14.5%
Planned pregnancy	Binary	Logistic regression	19.7%
Maternal anaemia	Ordinal (3 categories)	Logistic regression	20.5%
Iron supplementation	Binary	Logistic regression	36.3%
Vitamin mix supplementation	Binary	Logistic regression	67.1%
Vitamin B9 supplementation	Binary	Logistic regression	69.2%

All variables were included in the linear predictor of all imputation models, except the variable concerned by imputation.

Supplementary Table 2. Sensitivity analyses on multivariate associations between familial characteristics and timing of folic acid supplementation in reference to no supplementation.

	Exclusion of unplanned pregnancy or fertility treatment (n=10,912)		Main dataset (n=14,157)		Multiple imputations with 5 independent datasets (n=16,808)	
	Non-weighted analyses		Weighted analyses		Non-weighted analyses	
	Periconceptional supplementation	Late supplementation only	Periconceptional supplementation	Late supplementation only	Periconceptional supplementation	Late supplementation only
Age at first delivery						
< 25 years	0.76 [0.67 - 0.87]	1.11 [0.92 - 1.35]	0.71 [0.62 - 0.82]	1.11 [0.92 - 1.35]	0.75 [0.67 ; 0.83]	1.02 [0.87 ; 1.19]
25-29 years	1 [Ref]	1 [Ref]	1 [Ref]	1 [Ref]	1 [Ref]	1 [Ref]
30-34 years	1.16 [1.04 - 1.29]	1.21 [1.01 - 1.44]	1.21 [1.07 - 1.36]	1.40 [1.16 - 1.69]	1.24 [1.13 ; 1.36]	1.29 [1.11 ; 1.49]
≥ 35 years	1.20 [1.00 - 1.43]	1.34 [1.00 - 1.78]	1.21 [1.00 - 1.46]	1.45 [1.09 - 1.94]	1.31 [1.13 ; 1.50]	1.33 [1.05 ; 1.67]
Birth order						
First child	1 [Ref]	1 [Ref]	1 [Ref]	1 [Ref]	1 [Ref]	1 [Ref]
Second child	0.63 [0.57 - 0.70]	0.93 [0.80 - 1.09]	0.63 [0.57 - 0.70]	0.92 [0.78 - 1.08]	0.66 [0.61 ; 0.72]	0.93 [0.82 ; 1.06]
Third child	0.63 [0.54 - 0.73]	0.89 [0.71 - 1.12]	0.60 [0.52 - 0.71]	1.00 [0.80 - 1.24]	0.61 [0.54 ; 0.69]	0.96 [0.81 ; 1.15]
Fourth child or more	0.42 [0.31 - 0.56]	0.88 [0.62 - 1.26]	0.37 [0.28 - 0.48]	0.86 [0.63 - 1.17]	0.42 [0.34 ; 0.52]	0.90 [0.70 ; 1.16]
Family composition						
Traditional	1 [Ref]	1 [Ref]	1 [Ref]	1 [Ref]	1 [Ref]	1 [Ref]
Single-parenthood	0.86 [0.62 - 1.19]	0.79 [0.50 - 1.23]	0.60 [0.44 - 0.84]	0.70 [0.48 - 1.02]	0.75 [0.60 ; 0.93]	0.94 [0.74 ; 1.21]
Stepfamily	1.17 [0.97 - 1.41]	1.28 [0.98 - 1.66]	1.13 [0.93 - 1.36]	1.16 [0.90 - 1.50]	1.20 [1.03 ; 1.39]	1.18 [0.94 ; 1.47]
Migration						
Native French	1 [Ref]	1 [Ref]	1 [Ref]	1 [Ref]	1 [Ref]	1 [Ref]
Immigrant	0.96 [0.82 - 1.13]	1.24 [0.98 - 1.57]	0.90 [0.76 - 1.05]	1.37 [1.10 - 1.70]	0.88 [0.77 ; 1.00]	1.26 [1.02 ; 1.56]
Descendant of immigrant	0.88 [0.78 - 1.00]	0.98 [0.81 - 1.18]	0.95 [0.83 - 1.08]	1.12 [0.92 - 1.35]	0.94 [0.84 ; 1.04]	1.10 [0.93 ; 1.3]
Education level						
< Secondary school	0.46 [0.35 - 0.59]	0.71 [0.49 - 1.02]	0.49 [0.38 - 0.63]	0.74 [0.54 - 1.03]	0.43 [0.35 ; 0.53]	0.78 [0.60 ; 1.02]
Secondary school	0.47 [0.39 - 0.57]	0.69 [0.52 - 0.92]	0.55 [0.45 - 0.68]	0.58 [0.44 - 0.78]	0.47 [0.40 ; 0.55]	0.70 [0.55 ; 0.89]
High school	0.68 [0.58 - 0.80]	0.74 [0.57 - 0.95]	0.68 [0.57 - 0.80]	0.73 [0.56 - 0.95]	0.65 [0.57 ; 0.74]	0.77 [0.62 ; 0.97]
2-y university degree	0.76 [0.67 - 0.87]	0.90 [0.72 - 1.13]	0.77 [0.66 - 0.89]	0.82 [0.64 - 1.04]	0.74 [0.66 ; 0.83]	0.88 [0.72 ; 1.07]
3-y university degree	0.89 [0.78 - 1.02]	0.95 [0.76 - 1.20]	0.94 [0.81 - 1.10]	0.88 [0.69 - 1.13]	0.88 [0.78 ; 0.99]	0.92 [0.75 ; 1.13]

≥ 5-y university degree	1 [Ref]	1 [Ref]	1 [Ref]	1 [Ref]	1 [Ref]	1 [Ref]
Employment status						
Employed	1 [Ref]	1 [Ref]	1 [Ref]	1 [Ref]	1 [Ref]	1 [Ref]
Retired/disability/unemployed	0.89 [0.76 - 1.03]	1.21 [0.98 - 1.50]	0.84 [0.71 - 0.99]	1.14 [0.91 - 1.41]	0.84 [0.74 ; 0.95]	1.13 [0.95 ; 1.34]
Housewife/parental leave	0.93 [0.76 - 1.13]	0.87 [0.66 - 1.15]	0.78 [0.65 - 0.95]	1.16 [0.92 - 1.46]	0.84 [0.72 ; 0.98]	0.94 [0.77 ; 1.14]
Other	0.82 [0.57 - 1.17]	0.67 [0.37 - 1.21]	0.75 [0.52 - 1.08]	0.55 [0.30 - 0.99]	0.76 [0.59 ; 0.99]	0.72 [0.48 ; 1.07]
Student	1.31 [1.03 - 1.66]	1.09 [0.73 - 1.62]	0.94 [0.74 - 1.21]	0.85 [0.57 - 1.28]	1.13 [0.92 ; 1.37]	1.00 [0.73 ; 1.38]
Family income						
< €1,500	0.62 [0.50 - 0.77]	1.04 [0.78 - 1.40]	0.57 [0.46 - 0.71]	0.77 [0.59 - 1.02]	0.62 [0.52 ; 0.76]	0.86 [0.68 ; 1.09]
€1,501-2,300	0.75 [0.64 - 0.87]	1.13 [0.90 - 1.41]	0.79 [0.68 - 0.93]	1.06 [0.86 - 1.32]	0.80 [0.70 ; 0.92]	1.01 [0.85 ; 1.21]
€2,301-3,000	1 [Ref]	1 [Ref]	1 [Ref]	1 [Ref]	1 [Ref]	1 [Ref]
€3,001-4,000	1.06 [0.94 - 1.19]	1.17 [0.97 - 1.42]	1.02 [0.89 - 1.16]	1.00 [0.82 - 1.22]	1.07 [0.97 ; 1.19]	1.02 [0.86 ; 1.20]
€4,001-5,000	1.11 [0.95 - 1.29]	1.03 [0.79 - 1.35]	1.15 [0.97 - 1.37]	0.99 [0.75 - 1.31]	1.13 [0.98 ; 1.30]	0.94 [0.75 ; 1.17]
> €5,000	1.27 [1.06 - 1.51]	1.09 [0.81 - 1.46]	1.30 [1.07 - 1.58]	0.87 [0.63 - 1.20]	1.32 [1.12 ; 1.54]	0.86 [0.67 ; 1.11]
Pre-pregnancy BMI						
< 18.5 kg/m^2	1.07 [0.91 - 1.27]	1.09 [0.84 - 1.42]	1.05 [0.87 - 1.26]	1.20 [0.93 - 1.55]	1.04 [0.91 ; 1.19]	1.30 [1.07 ; 1.59]
18.5-24.9 kg/m^2	1 [Ref]	1 [Ref]	1 [Ref]	1 [Ref]	1 [Ref]	1 [Ref]
25.0-29.9 kg/m^2	0.82 [0.72 - 0.92]	0.91 [0.75 - 1.10]	0.76 [0.67 - 0.87]	0.88 [0.72 - 1.06]	0.82 [0.74 ; 0.90]	0.94 [0.81 ; 1.09]
30 kg/m^2 or more	0.65 [0.55 - 0.78]	1.00 [0.79 - 1.26]	0.69 [0.58 - 0.82]	1.01 [0.80 - 1.27]	0.75 [0.66 ; 0.86]	0.97 [0.80 ; 1.16]
Smoking status						
Never smoked	1 [Ref]	1 [Ref]	1 [Ref]	1 [Ref]	1 [Ref]	1 [Ref]
Only before pregnancy	1.12 [1.01 - 1.24]	1.10 [0.93 - 1.30]	1.10 [0.98 - 1.23]	1.09 [0.91 - 1.30]	1.10 [1.01 ; 1.20]	1.02 [0.89 ; 1.17]
Only during early pregnancy	0.79 [0.62 - 1.01]	1.15 [0.82 - 1.62]	0.69 [0.53 - 0.89]	0.93 [0.65 - 1.33]	0.78 [0.64 ; 0.94]	1.07 [0.81 ; 1.40]
During the whole pregnancy	0.73 [0.64 - 0.85]	0.95 [0.77 - 1.16]	0.71 [0.61 - 0.83]	0.95 [0.77 - 1.16]	0.70 [0.62 ; 0.79]	0.83 [0.70 ; 0.97]

Values are adjusted OR [95% CI]. Multivariate multinomial logistic regression, also adjusted for maternal region of residence, size of maternity unit, and wave of recruitment.
